# Evaluation of feedback methods for improved detection of hindlimb lameness in horses among riding instructors and trainers

**DOI:** 10.3389/fvets.2022.992954

**Published:** 2022-10-10

**Authors:** Anna Leclercq, Anna Byström, Maja Söderlind, Elisabeth Persson, Marie Rhodin, Maria Terese Engell, Elin Hernlund

**Affiliations:** ^1^Department of Anatomy, Physiology and Biochemistry, Swedish University of Agricultural Sciences, Uppsala, Sweden; ^2^Department of Companion Animal Clinical Sciences, Norwegian University of Life Sciences, Oslo, Norway

**Keywords:** perceptual learning, lameness detection, visual assessment, lameness, equine

## Abstract

Lameness, a wellknown issue in sport horses, impedes performance and impairs welfare. Early detection of lameness is essential for horses to receive needed treatment, but detection of hindlimb lameness is challenging. Riding instructors and trainers observe horses in motion in their daily work and could contribute to more efficient lameness detection. In this cross-sectional and prospective study, we evaluated the ability of riding instructors and trainers to assess hindlimb lameness. We also evaluated different feedback methods for improved lameness detection. For the cross-sectional part, *n* = 64 riding instructors and trainers of varying level and *n* = 23 high-level trainers were shown 13 videos of trotting horses, lameness degree: 0–3.5 (test 1) and tasked with classifying the horses as sound, left hindlimb lame, or right hindlimb lame. For the prospective part, the riding instructors and trainers of varying levels were randomly allocated to three different groups (a, b, c) and given 14 days of feedback-based, computer-aided training in identifying hindlimb lameness, where they assessed 13 videos (of which three were repeated from test 1) of horses trotting in a straight line. Participants in groups a-c received different feedback after each video (group a: correct answer and re-viewing of video at full and 65% speed; group b: correct answer, re-viewing of video at full and 65% speed, narrator providing explanations; group c: correct answer and re-viewing of video at full speed). After computer-aided training, the participants were again subjected to the video test (test 2). Participants also provided background information regarding level of training etcetera. Effects of participants' background on results were analyzed using analysis of variance, and effects of the different feedback methods were analyzed using generalized estimation equations. On test 1, 44% (group a), 48% (b), 46% (c), and 47% (high-level trainers) of horses were correctly classified. Group a participants significantly improved their test score, both with (*p* < 0.0001) and without (*p* = 0.0086) inclusion of repeated videos. For group c, significant improvement was only seen with inclusion of repeated videos (*p* = 0.041). For group b, no significant improvement was seen (*p* = 0.51). Although test 2 scores were low, computer-aided training may be useful for improving hindlimb lameness detection.

## Introduction

Pathology of the locomotor apparatus in horses is a common reason for owners to seek veterinary advice (1). Orthopedic disease is also one of the most common causes of euthanasia of horses (2). The clinical sign most commonly associated with diseases of the locomotor system in horses is lameness, but visual lameness detection is recognized as a major challenge by equine veterinary practitioners and subjective assessment is unreliable for evaluation of mildly lame horses (3). In particular, correct classification of hindlimb lameness appears to be more challenging than classification of forelimb lameness (3–5).

Although veterinarians are responsible for diagnosing and treating horses with orthopedic disease, the owner must first become aware of the problem, or be encouraged to seek veterinary advice by an equine professional such as a trainer, in order for these horses to receive veterinary care as needed. Interestingly, a large proportion of horses perceived as healthy by their owners have been judged as lame when subjectively evaluated by veterinarians (6, 7). Furthermore, a study using objective gait analysis found that >70% of 222 “owner-sound” horses presented with movement asymmetries above established thresholds (8). Although there are uncertainties regarding the cause of movement asymmetries (8, 9), the above findings may imply that horses with painful processes are in some cases ridden as though sound, which would pose a severe threat to animal welfare.

Perceptual learning can be explained as experience-induced improvement in the ability to recognize key stimuli that are of critical importance for differentiating between classifications (10). When studying hindlimb lameness, critical key stimuli can be translated into typical visual signs of lameness, e.g., differences in the vertical displacement of the sacrum (11). Potential of computer-based learning tools to improve identification of lameness has been demonstrated (12, 13), and it has been reasoned that computer-aided learning could be a complementary tool in training veterinary students (13). In a recent study, veterinary students were given a web-based learning tool showing animations of horses and tasked with classifying them as sound or lame (and, in case of lameness, to specify the limb) (12). The participants received feedback (the correct answer was displayed after each submitted reply), and the difficulty level was increased progressively. It was concluded that the ability of the participants for correct classification of horses with mild lameness improved significantly after < 2 h of training (12). However, that study did not investigate whether this improvement increased the ability of the participants to detect lameness in live horses.

Riders frequently attend lessons held by riding instructors or trainers. Riding instructors and trainers typically observe several different horses in motion during their daily work, and thus could potentially play a key role in detecting early signs of orthopedic disease in horses, and thereby help to ensure timely veterinary intervention. To our knowledge, the ability of trainers and riding instructors to visually detect lameness in horses has not been scientifically studied. The aims of this study were to (I) investigate the ability of riding instructors and high-level trainers to detect and classify hindlimb lameness in horses (cross-sectional part), and (II) to compare differences in learning outcomes between three different feedback methods in a computer-aided training program to improve the ability to detect hindlimb lameness (prospective part). The effects of different factors, such as previous experience and self-rated ability, on baseline performance (cross-sectional and prospective parts) and improvement (prospective part) were also evaluated.

## Materials and methods

### Study design

**Figure 1:**
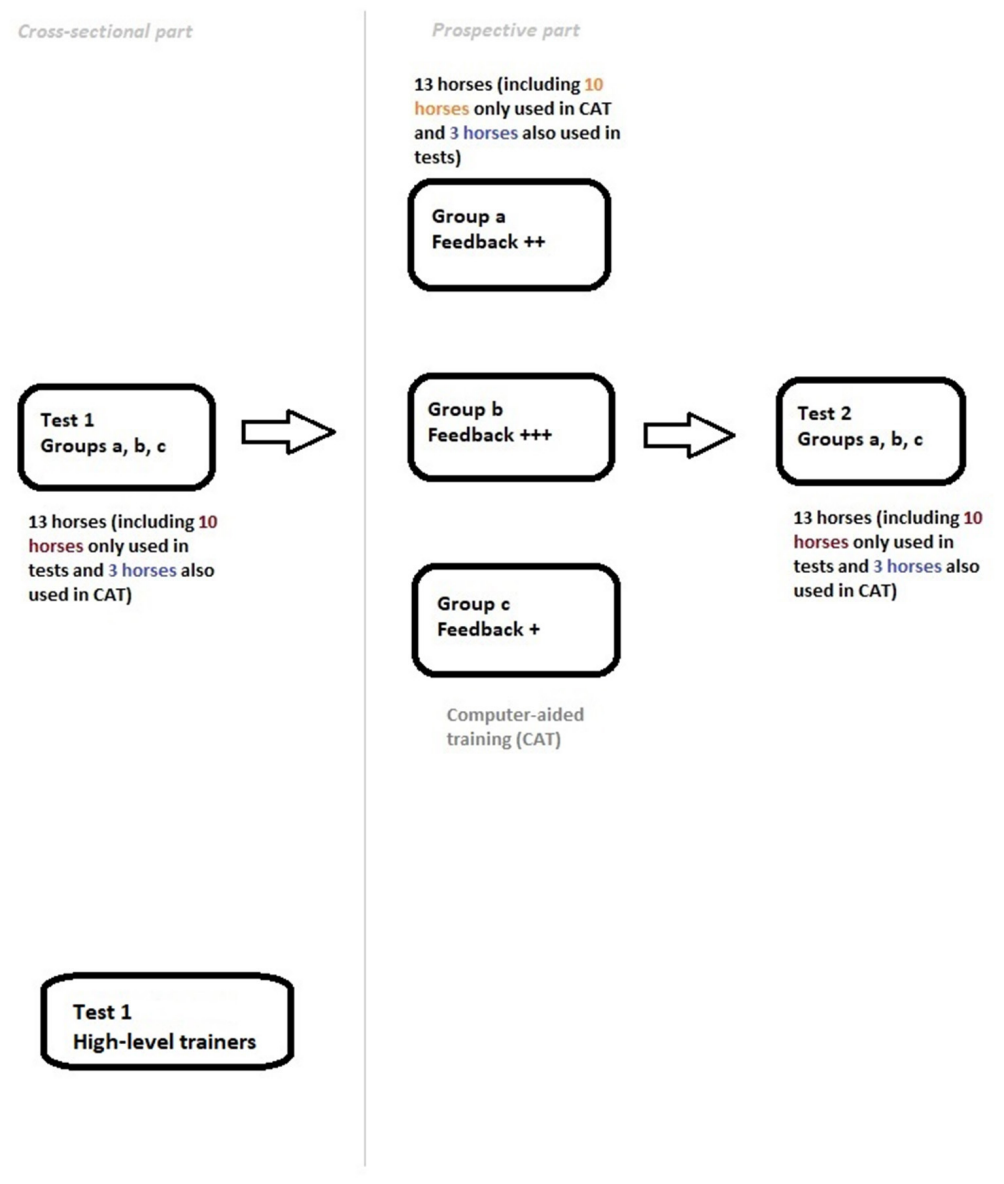
Simplified outline of the study.

The study comprised a cross-sectional part and a prospective part. The cross-sectional part consisted of a diagnostic test in which riding instructors and trainers were asked to assess hindlimb lameness in horses shown in videos. They were also asked a number of questions regarding their training level, previous experience, etc., as described below. In the prospective part of the study, riding instructors and trainers of varying level were given 2 weeks of computer-aided training (CAT) involving three randomly assigned feedback methods. The feedback methods were evaluated by comparing baseline test performance to post-CAT test performance. See Figure 1 below for simplified outline of the study.

#### Participants

Two groups of riding instructors and trainers participated in the study. A group of riding instructors and trainers of varying levels were recruited *via* an announcement on the website of the Swedish Equestrian Federation (SEF). A group of high-level trainers (all level A, as defined by the SEF) attending a further education lecture hosted by SEF were also asked to participate and, for practical reasons, were only included in the cross-sectional part of the study. Participants were included if they had formal training and SEF certification as riding instructors or equestrian trainers. Participants in the group of instructors and trainers of varying levels were excluded from both parts of the study if they did not complete both tests and the CAT. Written informed consent was obtained from all included participants.

### Cross-sectional part

The participants were subjected to a video-based test to assess their ability to identify hindlimb lameness (test 1, see Section Test 1 and test 2). Participants also included in the prospective part (i.e., riding instructors and trainers of varying level) performed this test within the online learning platform used for the CAT (see Section Computer-aided training). For the group of high-level trainers, all videos used in test 1 (13 in total) were displayed twice to all participants simultaneously, on a large screen instead of individual computers, and answers were submitted individually using a paper form. Possible answers were “sound,” “right hind lame,” and “left hind lame,” i.e., random guess accuracy was 1/3. All participants were also instructed to complete a questionnaire containing questions with preset answer options, regarding: level of training, years of experience, level of riders taught, previous experience of lameness assessment, self-rated ability to assess hindlimb lameness, previous training in lameness detection, and approximate number of horses seen at work on a weekly basis. In the same questionnaire, they were also asked to state (in free text) how many times during the previous 6 months they had interrupted a lesson due to abnormal gait in a horse or had been asked by a student to assess whether a horse was lame. High-level trainers were also asked to state their opinion on the importance of trainers being able to detect lameness, and whether they found the video quality sufficient. Participants in the prospective part of the study were asked similar questions in the post-study form at the end of the CAT period (see below). The riding instructors and trainers of varying level completed the questionnaire on the digital learning platform, and for the high-level trainers, a paper form was used.

### Prospective feedback study part

The prospective part of the study was conducted in conjunction with a lecture (further education) for riding instructors and trainers organized by the SEF. The course consisted of three online live sessions 1 week apart, which included lectures on topics other than lameness detection, and participants were expected to practice individually between the live sessions. Participants were randomly divided into three groups (a, b, c) using Microsoft Excel (2016), and were sent an email link inviting them to register on a digital learning platform (Canvas, Instructure, London, UK). Groups a, b, and c received separate links, and thus the participants only had access to material intended for their assigned group. Each participant was instructed to create a personal account on the platform. On day one of the study, participants were instructed to perform a diagnostic, video-based test as described above and they were then subjected to individual CAT. During the CAT, all participants had access to the same video-based practice quiz, but received different feedback after each video depending on group, with group b receiving the most detailed feedback and group c the least detailed feedback (see below). Group b also had access to an instructional film as part of their CAT. All participants had unlimited access to their respective CAT modules during the 2-week practice period. On day 14, all participants were again subjected to a diagnostic, video-based test (test 2), which was identical to test 1. After completing test 2, the participants were asked to complete a second digital questionnaire (referred to as post-study form) comprising six questions with preset answer options, on: the perceived difficulty of the videos, whether the CAT had improved the ability of the participant to assess hindlimb lameness, the quality of the CAT, the quality of the videos, whether the participant would recommend the CAT to others, and the importance of trainers being able to detect lameness. The participants were also given the opportunity to submit general feedback (in free text) on the CAT and on the study in general. All video editing was performed in Camtasia 2021 (Techsmith, Okemos, MI, USA).

#### Test 1 and test 2

**Figure 2:**
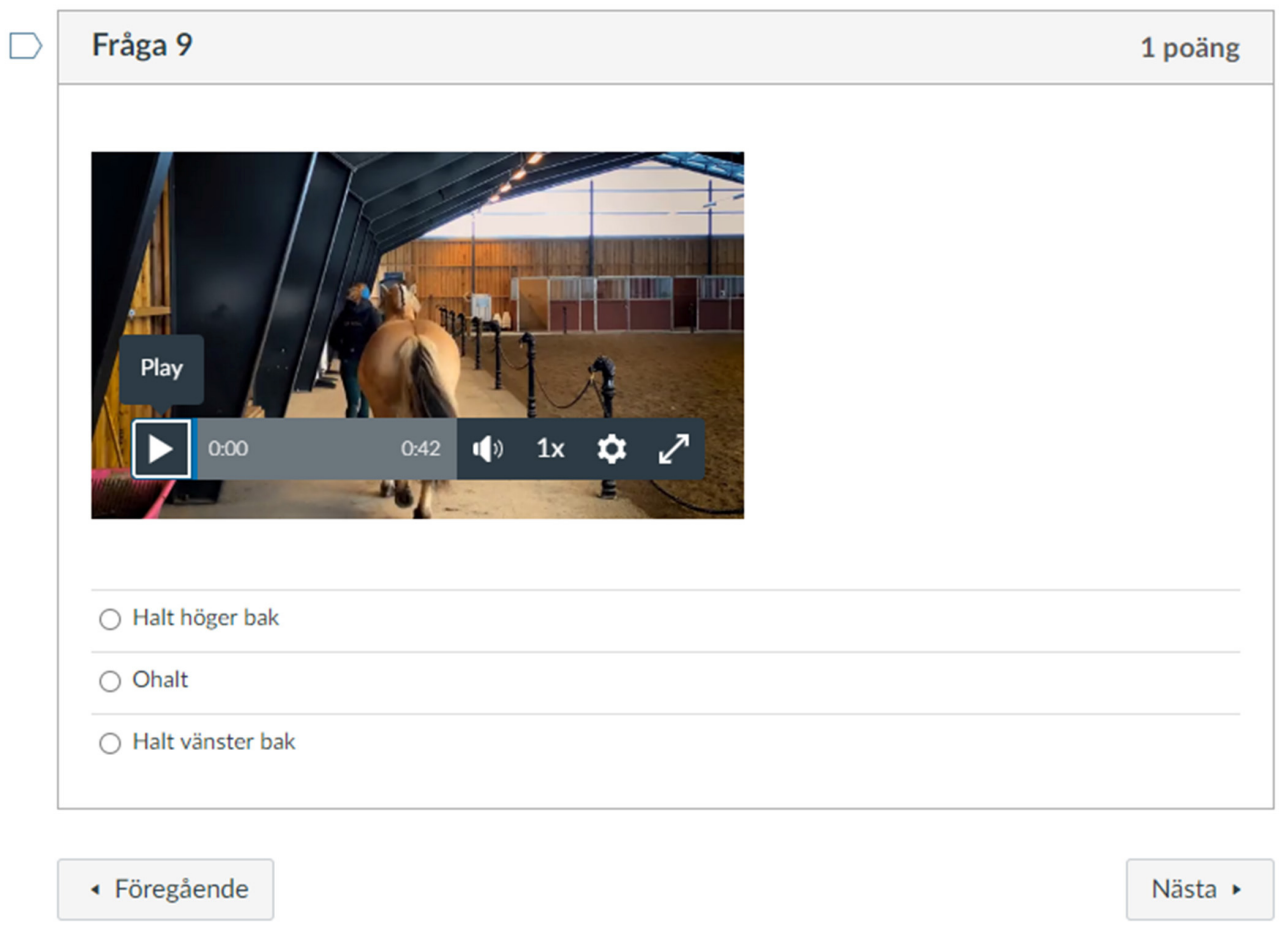
Print screen from the digital learning platform, showing still picture from one of the videos used in test 1 and 2.

Tests 1 and 2 both contained the same 13 videos, each of which showed a different horse trotting in a straight line (see Figure 2 below). All horses were shown trotting both toward and away from the camera. Three of the horses were sound, six were left hind lame, and four were right hind lame. The lame horses were subjectively judged as mildly to moderately lame (grade 0.5–3.5, on a 0–5 ordinal lameness scale) by two experienced veterinarians (authors EH and ME). Audio from the video recordings was provided for seven (video numbers 2, 4, 5, 6, 7, 10, and 13) of the videos, but removed from the remaining six because of disturbing background noises. Eleven horses were led from the left side, while the remaining two (video numbers 3 and 12) horses were led from both sides (alternated by handler). Seven horses (video numbers 2, 8, 9, 10, 11, 12, 13) were shown on a hard surface, while the remaining six horses were shown on a soft surface. All horses were evaluated using objective gait analysis to confirm lameness. Six of the horses were warmbloods, two were coldblooded trotters, and two were Norwegian fjord horses. The remaining three horses comprised one pony, one Icelandic horse, and one quarter horse. The horses were privately owned, and informed consent was obtained from the owners. For each video, the participants were asked to classify the horses as sound, right hind lame, or left hind lame (i.e., random guess accuracy was 1/3). Total score (X/13) was shown to each individual after completion of each of the tests, but correct answers were not displayed to participants during the study period. Tests 1 and 2 could only be opened and completed one time each, and videos were locked for viewing before and after completion of each test (see also Table 1).

**Table 1:** Ratios of participants who provided a correct answer for each video, categorized by group (a, b and c) and test (1, 2).

**Video number**	**Test 1, group a**	**Test 2, group a**	**Test 1, group b**	**Test 2, group b**	**Test 1, group c**	**Test 2, group c**	Lameness grade^*^	**Repeated video (Yes/No)**
1	0.39	0.56	0.83	0.70	0.48	0.61	1–1.5	No
2	0.28	0.67	0.39	0.65	0.09	0.70	0.5	Yes
3	0.61	0.83	0.65	0.78	0.43	0.87	1–1.5	Yes
4	0.28	0.50	0.26	0.26	0.39	0.52	1.5–2	No
5	0.39	0.83	0.70	0.78	0.70	0.83	1.5–2	No
6	0.61	0.50	0.70	0.65	0.74	0.61	0	No
7	0.39	0.83	0.35	0.39	0.43	0.39	1.5	No
8	0.17	0.11	0.17	0.30	0.04	0.26	1.5	No
9	0.50	0.67	0.57	0.48	0.57	0.57	2	No
10	0.50	0.83	0.30	0.43	0.52	0.65	0	Yes
11	0.50	0.72	0.52	0.35	0.65	0.30	3.5	No
12	0.50	0.61	0.48	0.48	0.35	0.52	1.5	No
13	0.61	0.44	0.39	0.39	0.57	0.22	0	No
Mean, all videos	0.44	0.62	0.48	0.51	0.46	0.54		
Mean, non-repeated videos	0.43	0.57	0.50	0.48	0.49	0.48		

Videos 2, 3, and 10 were repeated videos. Mean test scores (expressed as ratios, categorized by group and test, both total and with exclusion of repeated videos) are presented in the bottom lines. Horses in videos 6, 10, and 13 were sound.

* Subjectively graded by EH and MTE, using a 0-5 ordinal lameness scale.

#### Computer-aided training

The varying levels participants had free access to the CAT for a period of 14 days, i.e., between tests 1 and 2. The CAT included a digital quiz, intended for practicing, that comprised 13 videos similar to those included in tests 1 and 2. Five of the horses in these videos were right hind lame, five were left hind lame, and three were sound. Subjective lameness grade on a 0–5 ordinal scale ranged between 0.5 and 3 (assigned by EH and MTE). Audio from the video recordings was provided for nine of the videos, but removed from the remaining four because of disturbing background noises. All horses except one were led from the left side. In the remaining video, the side was alternated by the handler. Twelve of the horses were shown on a hard surface, and one was shown on a soft surface. Ten of the videos used in the CAT were unique, while three were also used in tests 1 and 2 (referred to hereafter as ‘repeated videos'). The participants were asked to classify each horse as sound, right hind lame, or left hind lame. One completion of the CAT was defined as classifying ≥8 different videos during one coherent session. After each video, feedback was displayed. For group a, the feedback consisted of the correct answer in writing (displayed at the beginning) and re-viewing of the video played at full speed and at 65% of full speed. Group b received similar feedback, but with the addition of a narrator explaining the adaptive motion (in case of lameness) in detail. In re-viewing at 65% of full speed, the speed was decreased further to 20–30% of full speed for 2–4 trotting strides. Group b was also given access to a film (15 minutes long) explaining important visual landmarks (such as the mid-pelvis and tubera coxarum) and the motion that should be evaluated for lameness detection (vertical displacement of the sacrum, total vertical movement of the tubera coxarum, and pelvic rotation) (14–16). This film included demonstrations using a pelvic model, three sound horses, and two hindlimb lame horses trotting in straight line. Group c was only allowed re-viewing of each video at full speed, with the correct answer (shown at the start) as feedback.

### Statistical analysis

For the riding instructors and trainers of varying level participating in the prospective and cross-sectional study parts, participant data were extracted from the digital learning platform and descriptive statistics were calculated. Unfortunately, logs from the digital learning platform revealed that 23 participants had accidentally performed the CAT at least once (≥8 videos) before entering test 1 (group a: *n* = 8, group b: *n* = 15, group c: *n* = 0), and this was therefore included as a variable in the dataset (trained before test 1 yes/no). For the high-level trainers who only participated in the cross-sectional part, responses were manually extracted from the response sheets and descriptive statistics were calculated. The two datasets were then merged. Statistical analysis was performed in R version 4.1.2.

Effects of responses to the questionnaire questions with preset answer options (addressing background, experience, level of training, etc., as detailed above) on the number of correct answers in test 1 (i.e., one datapoint per participant) were evaluated using analysis of variance (ANOVA) in simple linear models, based on the complete dataset (R package lme4). One model per question was created. Due to a small number of responses per category, responses to the question “years of experience” were condensed to ≤ 10 years or >10 years (original alternatives: 1–3, 3–5, 5–10, and >10 years). Normality of model residuals was confirmed using QQ-plots.

Effects of responses to the various questionnaire questions on the change in number of correct responses between test 1 and test 2 were evaluated using the same model strategy as above, but including data only for participants in the prospective part of the study. An evaluation was also performed on whether participants who inadvertently trained before test 1 performed better in test 1, and whether this affected the change in number of correct answers between the two tests, again using the same model strategy.

Effects of the CAT, including the three different types of feedback (groups a-c), were evaluated using generalized estimation equations (R package gee), applied to data from the prospective part of the study. The dataset consisted of responses to each of the 13 videos in the respective tests, with correct/incorrect answers coded as 1/0. Three different models were created, all with individual included as a random factor. In the first model, the interaction between group (a-c) and test (1, 2) was evaluated, i.e., differences between test occasions within each group and differences between groups on each test occasion. The same model was also made including only data for videos that were not included in the training. Finally, the interaction between whether the video was a repeated video (yes/no) and test (1, 2) was evaluated in the same manner (including all data). Correlation structure was set to independence in all cases, as there were only two time points for each individual. The estimated scaling parameter was close to one in all models (differing only at the third decimal).

For all models, least square means and contrasts between categories were calculated for fixed factors with a significant *P*-value (R package emmeans). The limit for statistical significance was set to *p* < 0.05 in all analyses. Correction for multiple comparisons was applied to comparisons between variable categories (e.g., comparing groups a-c) within each model, using the Tukey method (as implemented in emmeans). Confidence interval for odds ratios (OR) was manually calculated. For datasets used in statistical analyses, see Supplementary material.

## Results

### Participants

A total of 76 trainers and riding instructors of varying levels initially registered for participation. Four participants withdrew before onset of the study and did not participate. Eight participants were excluded from the questionnaire and test results, due to failure to carry out test 1, the CAT, or test 2. Hence, 64 riding instructors and trainers (group a: *n* = 18, group b: *n* = 23, group c: *n* = 23) completed test 1, CAT, and test 2, and were included in the cross-sectional and prospective parts of the study. Twenty-three high-level trainers completed test 1 and the questionnaire, and were thus included in the cross-sectional part of the study.

### Descriptive statistics

#### Prospective study part (riding instructors and trainers of varying levels)

Ratios of participants (overall) who provided the correct answer for each video, as well as mean scores for tests 1 and 2, are presented in Table 1. Overall, 46% of the horses (6.0/13) (or 43% (5.6/13) on exclusion of test 1 scores from participants who completed the CAT before test 1) were correctly classified in test 1 and 56% (7.2/13) in test 2. Median (range) for number of completions of CAT between submission of test 1 and test 2 for each group was as follows: group a: 4 (1-9), group b: 3 (1-7), group c: 4 (1-18).

After completion of tests 1 and 2, all included participants except two completed the post-study form. Overall, 87% of responders considered the video quality to be sufficient. Half of the responders (50%) in group a stated that they believed the CAT was “satisfactory,” while 28% stated that the CAT was “good and contributing to the learning process.” The remaining 22% found the CAT “non-satisfactory.” In group b, a large majority (86%) considered the CAT to be “good and contributing to the learning process.” Only 5% (one participant) found the CAT “non-satisfactory,” while the remaining 9% found it “satisfactory.” In group c, a large majority (73%) considered the CAT to be “non-satisfactory” and only 14% found it “good and contributing to the learning process.” A further 9% found it “satisfactory” and 5% (one participant) failed to answer this question. The percentage of responders stating that they would recommend the CAT to others was 100% for group a, 91% for group b, and 91% for group c.

#### High-level trainers

The group of high-level trainers, who only participated in the cross-sectional part of the study, on average correctly classified 47% (6.1/13) of horses in test 1.

#### Questionnaire on level of training, experience, etc.

##### Riding instructors and trainers of varying levels

As mentioned, participants in the prospective part of the study (riding instructors and trainers of varying levels) who failed to complete test 1, CAT, or test 2 were excluded from analysis of the questionnaire results. One participant in group c failed to complete the questionnaire, but was still included in the other results.

When asked how many times during the previous 6 months they had interrupted a lesson due to abnormal gait in a horse or been asked by a student to assess whether a horse was lame, 86% of included participants (overall) reported being involved in one or both situations at least once. For descriptive results on the remaining questions (i.e., questions with preset answer options), see Table 2.

**Table 2:** Descriptive results on questionnaire responses, expressed as a percentage of responding participants in each group (one missing for group c).

**Question**	**Group a**	**Group b**	**Group c**	**High-level trainers**
**Highest level of training:**
Riding instructor^**^	33%	17%	5%	0%
Trainer Level A^**^	6%	0%	5%	100%
Trainer Level B^**^	6%	13%	23%	0%
Trainer Level C^**^	56%	70%	68%	0%
**Years of experience:**
<10	11%	17%	18%	0%
>10	89%	83%	82%	100%
**Level of majority of riders taught:**
Low	83%	70%	64%	17%^*^
Middle	17%	26%	32%	70%^*^
Advanced	0%	4%	5%	13%^*^
**Previous experience of lameness assessment (self-rated)**
None	22%	26%	27%	4%
Some	67%	70%	64%	74%
Advanced	11%	4%	9%	22%
**Ability to assess hindlimb lameness (self-rated)**
None	11%	9%	9%	0%
Some	83%	87%	82%	78%
Advanced	6%	4%	9%	22%
**Previous training in lameness assessment**
Yes	6%	0%	5%	9^***%^
No	94%	100%	95%	87^***%^
**Number of horses seen weekly**
<10	28%	26%	18%	13^***%^
10–20	50%	30%	27%	22^***%^
>20	22%	43%	55%	61^***%^
**Number of respondents**	18	23	22	23

* Among the high-level trainers, some ticked multiple answers (always including the option “middle”) to this question (instead of only one), possible due to their questionnaire being in paper form. These participants were regarded as having answered “middle”.

** As defined by the SEF.

*** One participant in this group failed to answer this question, but answered the remaining questions.

##### High-level trainers

According to the questionnaire responses from the high-level trainers only participating in the cross-sectional part of the study, 83% found the video quality sufficient. When asked how many times during the previous 6 months they had interrupted a lesson due to abnormal gait in a horse or had been asked by a student to assess whether a horse was lame, 65% of participants reported being involved in one or both situations at least once. For descriptive results on the remaining questions, see Table 2.

### Analytical statistics, prospective and cross-sectional study parts

#### Effect of questionnaire responses and completion of CAT before test 1

Self-rated ability to assess hindlimb lameness significantly affected the score in test 1. Participants self-reporting “advanced” ability (mean score 7.56, 95% confidence interval (CI) 6.36–8.75) obtained significantly higher scores in test 1 than those self-reporting “some” ability (mean score 6.01, CI 5.59–6.44, *p* = 0.047) or “no” ability (mean score 4.83, CI 3.37–6.30, *p* = 0.015). No other questionnaire responses had any significant effect on test 1 score or change of score between test 1 and test 2. Participants in the prospective study part who had inadvertently completed the CAT before test 1 received significantly higher scores in test 1 than those who had not (mean score 6.65, CI 5.88–7.42 and mean score 5.66, CI 5.08–6.23, respectively, *p* = 0.043). However, change of score between test 1 and test 2 was not significantly affected (mean improvement: 0.61 (-0.44–1.66) and 1.49 (0.70–2.28), respectively, *p* = 0.19). For full details, see Supplementary material.

#### Effect of feed-back methods and repeated questions

There were no significant differences in scores between any of the feedback groups for test 1. For test 2, group a participants received significantly higher scores than group b participants (OR = 1.56, CI 1.10–2.20, *p* = 0.035). Comparison of differences in scores between test 1 and test 2 revealed that group a participants received significantly lower scores in test 1 than in test 2 (OR = 0.48, CI 0.33–0.70, *p* = 0.0001). This was also true for group c (OR = 0.72, CI 0.51–0.99, *p* = 0.041). For group b, there was no significant difference in scores between tests 1 and 2 (OR = 0.90, CI 0.65–1.24, *p* = 0.51).

On exclusion of the three repeated videos (videos 2, 3, and 10), which were used both in tests and in the CAT, there were no significant differences in scores between any of the groups in test 1 or test 2. Group a obtained significantly lower scores in test 1 than test 2 (OR = 0.57, CI 0.38–0.87, *p* = 0.0086). None of the other groups showed any significant difference in scores between tests 1 and 2 on exclusion of the repeated videos (group b: OR = 1.07, CI 0.74–1.55, *p* = 0.71; group c: OR = 1.035, CI 0.72–1.49, *p* = 0.85).

Overall, there was no significant score change between tests 1 and 2 when excluding repeated videos (OR = 0.89, CI 0.71–1.11, *p* = 0.29). When excluding videos only used in test 1 and test 2 (i.e., only including repeated videos), the scores in test 1 were, overall, significantly lower than the scores in test 2 (OR = 0.29, CI 0.19–0.45, *p* < 0.0001). In test 1, there was no significant difference in mean score between repeated videos and non-repeated videos (OR = 1.28, CI 0.92–1.77, *p* = 0.15). In test 2, mean score for non-repeated videos was significantly lower than that for repeated videos (OR = 0.42, CI 0.29–0.60, *p* < 0.0001).

Comparison of scores in test 1 and test 2 for each individual video revealed that all repeated videos were (overall) correctly classified significantly less frequently in test 1 than in test 2 (video 2: OR = 0.16, CI 0.08–0.35, *p* < 0.0001; video 3: OR = 0.27, CI 0.12–0.60, *p* = 0.0015; video 10: OR = 0.47, CI 0.23–0.95, *p* = 0.035). Among the non-repeated videos, only one (video 5) was significantly less likely to be correctly answered in test 1 than test 2 (OR = 0.36, CI 0.16–0.80, *p* = 0.013). Among the remaining videos, there were no statistically significant differences in scores between tests 1 and 2, although video 13 tended to be correctly classified more frequently in test 1 than in test 2 (*p* = 0.051) (see also Table 1). For full details, see Supplementary material.

## Discussion

In this study, three different feedback methods intended for improvement of hindlimb lameness classification were evaluated in a population of riding instructions and trainers of varying levels. To the best of our knowledge, this is the first study to investigate the ability of non-veterinary “horse professionals” to evaluate lameness. Among the three feedback methods studied here, the feedback received by group a (i.e., re-viewing videos in slow motion without receiving detailed verbal instructions and explanations) appeared to be most strongly associated with enhanced performance within the study timeframe, which was 2 weeks. Re-viewing the same video at many occasions also generally appeared to be beneficial for improvement of scores.

In the prospective part of the study, a significant improvement in baseline score was seen for two of the three groups compared, supporting previous findings that improved detection of lameness can be achieved with limited amounts of training (12). The feedback given to group a was associated with the largest improvement. Interestingly, group a was the only group for which a significant improvement in score was seen even with exclusion of the three repeated videos. This finding is of interest, as the repeated videos appear to have contributed to a major degree to the increase in average score across all groups. For group c, a (significant) improvement between test 1 and test 2 was seen only when the three repeated videos were included, showing that the improvement in this group was based on these videos. In group b, only a very slight improvement, which was not statistically significant, was seen. With exclusion of repeated videos, no improvement at all was seen, neither for group b nor c. However, group b received the highest average score in test 1, probably because a large proportion (15/23 or 65%) of participants in this group inadvertently completed the CAT before completion of test 1, which may have influenced the results (completion of CAT before test 1 was, overall, shown to influence test 1 results). Group b received the most detailed feedback and, although firm conclusions cannot be drawn, it is possible that participants were unable to process the provided feedback adequately due to ‘information overload', especially considering the short timeframe given for the CAT in this study. Listening to verbal feedback while watching the videos may also have distracted the participants and prohibited them from registering the visual input adequately.

As mentioned, the three repeated videos appeared to contribute strongly to the improvement in the scores; group a was the only group where improvement was seen on exclusion of these videos. As the correct answers were displayed during the CAT, it is unclear whether this improvement occurred because participants, by being allowed to see these videos many times, learned to interpret the lameness adaptation strategies of the horses in question, or whether they simply learned to recognize the videos (e.g., coat color of the horses or other stimuli not associated with lameness) and memorized the correct answers. The relative importance of the repeated videos for increasing test scores between test 1 and test 2 seemed to be greatest for group c. Group c received the least extensive feedback and consequently one round of the practice quiz took a shorter time, which is likely why some participants in this group tended to do more repetitions, and thus saw the repeated videos at a greater number of occasions. This could be why the repeated horses affected the change in test scores especially for this group, although it should be noted that group c was the only group where no participants had seen the CAT and thus the responses to the repeated videos in advance, which may have resulted in lower test 1 scores on these videos and thus, greater potential for improvement.

The fact that the feedback provided to group a was associated most strongly with improvement of score could be partly explained by the slow motion helping to overcome limitations posed by the human eye (17). Slowing down videos may have given the participants time to better “map out” the motion of the horse, e.g., by comparing the two sides of the horse or detecting time of hoof impact in relation to pelvic motion, and thus register key stimuli that are critical to be able to detect lameness. Becoming familiarized with the motion of the horse and learning when events take place in relation to each other may then help in registering these stimuli at full speed. Group b also received slow motion feedback, but showed the smallest improvement, which could be seen as conflicting. However, as mentioned, the extensive verbal feedback provided for group b in addition to the slow-motion re-viewing may have impaired the learning process by causing distraction and information overload. It should also be noted that the number of videos used in the study are low. Furthermore, the group of horses used in the videos was highly heterogeneous, and thus, firm conclusions about the efficacy of the feedback methods used cannot be drawn. Contradicting what might be anticipated, performance was not associated with level of training or years of experience. Test results for the high-level trainers were similar to the test 1 results for the trainers of varying levels, with 47% and 46% (or 43% with exclusion of scores from participants who inadvertently completed the CAT before test 1), respectively, of horses correctly classified. Neither did years of experience have any statistically significant effect on test 1 results or improvement. However, it has previously been shown that even among veterinarians (who, in contrast to riding instructors and trainers, are trained in lameness assessment and presumably expected to be able to detect lameness correctly), subjective lameness evaluation is unreliable (3). This indicates that experience and training are not necessarily correlated with a favorable outcome. Rather, for more reliable assessment of lameness, developing an ability to pick up on discriminant visual cues against a background of irrelevant ‘noise' is key. As mentioned, in a previous study where relevant landmarks were emphasized using animated videos, the ability of veterinary students to correctly classify lameness improved significantly after a short (<2 h) bout of training (11).

As revealed by the responses from the post-study form in the prospective part of the study, participants in group c, which received the least detailed feedback, were less satisfied with the CAT than participants in the other two groups. In light of this, it can be assumed that the limited information given to group c in the CAT contributed to participants believing that their learning process was not adequately supported, and that they were not gaining any new skills by completing the quiz. This could be expected to impact their motivation, and possibly also their performance, as motivation can have a positive impact on learning (18). However, participants in group B, which received the most extensive feedback, were most pleased with the training, but showed the smallest increase in test scores between test 1 and test 2. This suggests that there is no immediate correlation between perceived and actual effectiveness of short-term CAT.

Although a statistically significant improvement in baseline scores was seen for two of the three feedback groups in the prospective part of the study, it is important to emphasize that, on average, less than two-thirds of horses were correctly classified in test 2. A possible explanation for the low scores could be the limited time given for training, particularly as movement patterns can vary greatly among hindlimb lame horses (12), requiring the use of several different visual landmarks during assessment. The combined vertical movement and rotation of the pelvis also makes the visual impression more complex, and thus more difficult to process, compared with the more simple vertical movement of the head used when assessing forelimb lameness. Thus, lack of knowledge about what to look for when assessing lameness (e.g., only looking for vertical displacement of the sacrum and not the *tubera coxarum*) could also be part of the explanation for the low scores in test 2.

Some attention should also be drawn to the fact that the context in which riding instructors and trainers observe horses is generally different from the situations shown in our video material. During a typical riding lesson, horses are presented under a rider. It should be kept in mind that the presence of a rider can influence kinetic and kinematic parameters in trotting horses (19–21), which could influence perception of lameness. Although this study involves horses with lameness that could be detected during in-hand, straight line trot, lameness may in some cases only be detectable under a rider (22). Additionally, trainers may not solely observe horses trotting from a frontal and/or rear view. Conversely, they are likely to rather observe horses from the side and in different gaits (i.e., not only in trot). This is also usually the case during lameness exams when carried out by veterinarians. In our material, the horses were only shown in trot, and not from the side. Furthermore, audio recordings were (due to technical reasons) not included for all videos and thus, the participants could not always consider the sounds of the foot falls when choosing their answers, which would likely have been possible in a real-life situation. Taking the above in consideration, it could be considered unclear whether the scores seen here are reflective of the participants' actual ability to detect lameness in real life situations, and if the improvement seen could translate to an increased ability to detect lameness in horses during typical riding lessons.

On the other hand, even though a full lameness exam usually includes several steps where the horse is assessed under different circumstances, the condition shown in the video material (horses trotting up and down in a straight line) represents one of the most important components of a lameness exam (3). Furthermore, if lameness is suspected during a riding lesson, the trainer could, if deemed appropriate, instruct the rider to show the horse under different circumstances (e.g., ridden, unridden, from the side, from the front, and from the back) to optimize conditions.

## Conclusion

This study including a limited number of riding instructors and trainers, showed that lameness classification skills can be improved by providing even limited training. Furthermore, choice of learning method may potentially impact the outcome. However, background factors such as level of training and years of experience did not impact the score. Although the results were influenced by some participants accessing parts of the study in an incorrect order, slow-motion feedback without verbal explanations, as well as re-viewing of the same video several times, seemed to contribute to improvement of lameness detection. However, the group of horses used for the learning and test materials was small and heterogeneous, and the video material does not entirely represent the real-life situations that riding instructors and trainers can be expected to encounter in their daily work. Larger studies evaluating the effects of different learning methods on the ability to assess lameness in horses are needed to be able to draw conclusions.

## Data availability statement

The original contributions presented in the study are included in the article/Supplementary material, further inquiries can be directed to the corresponding author/s.

## Ethics statement

Ethical review and approval was not required for the study on human participants in accordance with the local legislation and institutional requirements. The patients/participants provided their written informed consent to participate in this study. Ethical review and approval was not required for the animal study because the horses used for the video material in the study were privately owned and no invasive procedures were carried out. The horses were filmed during routine lameness examinations and participation in other biomechanical studies. Thus, ethical permission was not needed according to local legislation. Written informed consent was obtained from the owners for the participation of their animals in this study.

## Author contributions

EH, ME, and AB contributed to conception and design of the study. ME, AL, and EH created learning material on the digital platform and executed data collection. AL and EH contributed to preparation of data and AB performed the statistical analysis. MS, AL, EH, AB, MR, and EP contributed with interpretation of data and results. AL wrote the first draft of the manuscript. All authors critically revised the manuscript and have approved the submitted version.

## Funding

This study was funded by the Swedish-Norwegian Foundation for Equine Research (project number, H-17-47-304). Ten percent of publication fees will be paid by the Swedish University of Agricultural Sciences.

## Conflict of interest

The authors declare that the research was conducted in the absence of any commercial or financial relationships that could be construed as a potential conflict of interest.

## Publisher's note

All claims expressed in this article are solely those of the authors and do not necessarily represent those of their affiliated organizations, or those of the publisher, the editors and the reviewers. Any product that may be evaluated in this article, or claim that may be made by its manufacturer, is not guaranteed or endorsed by the publisher.
